# Measuring the psychosocial burden in women with low-grade abnormal cervical cytology in the TOMBOLA trial: psychometric properties of the Process and Outcome Specific Measure (POSM)

**DOI:** 10.1186/s12955-014-0154-8

**Published:** 2014-11-12

**Authors:** Kieran Rothnie, Seonaidh C Cotton, Shona Fielding, Nicola M Gray, Julian Little, Margaret E Cruickshank, Leslie G Walker, Mark Avis, Linda Sharp, in association with the TOMBOLA group

**Affiliations:** Division of Applied Health Sciences, University of Aberdeen, Aberdeen, UK; School of Nursing & Midwifery, University of Dundee, Dundee, UK; Canada Research in Human Genome Epidemiology, Department of Epidemiology and Community Medicine, University of Ottawa, Ottawa, Canada; Obstetrics & Gynaecology, University of Aberdeen, Aberdeen, UK; The Postgraduate Medical Institute, University of Hull, in association with Hull York Medical School, Hull, UK; School of Health Sciences, University of Nottingham, Nottingham, UK; National Cancer Registry Ireland, Cork, Ireland

**Keywords:** Cervical intraepithelial neoplasia, Questionnaires, Factor analysis, Psychosocial distress

## Abstract

**Background:**

There is a need for an instrument to measure the psychosocial burden of receiving an abnormal cervical cytology result which can be used regardless of the clinical management women receive.

**Methods:**

3331 women completed the POSM as part of baseline psychosocial assessment in a trial of management of low grade cervical cytological abnormalities. Factor analysis and reliability assessment of the POSM were conducted.

**Results:**

Two factors were extracted from the POSM: Factor 1, containing items related to worry; and Factor 2 containing items relating to satisfaction with information and support received and change in the way women felt about themselves. Factor 1 had good reliability (Cronbach’s alpha 0.769), however reliability of the Factor 2 was poorer (0.482). Data collected at four subsequent time points demonstrated that the factor structure was stable over time.

**Conclusion:**

This study demonstrates the presence and reliability of a scale measuring worries within the POSM. This analysis will inform its future use in this population and in other related contexts.

**Electronic supplementary material:**

The online version of this article (doi:10.1186/s12955-014-0154-8) contains supplementary material, which is available to authorized users.

## Introduction

Almost 4 million cervical cytology tests are undertaken every year in the UK, and over 6% of these are reported as abnormal [1,2]. The majority of these are reported as showing low-grade abnormalities, i.e. borderline nuclear changes (BNC) or mild dyskaryosis.

It has been well established that receipt of an abnormal cervical cytology result can cause significant negative psychological consequences [3–12]. The adverse psychosocial sequelae relating to receipt of abnormal results and subsequent management represent the psychological cost of cervical screening. Several of these studies have investigated the effects of receiving an abnormal result on anxiety and depression. However, cervical screening may raise a range of other psychosocial issues for women, including worries about their health, sexual functioning, future fertility and the development of cervical cancer [13].

At least two measures [14–16] have been developed for assessing the burden of psychosocial sequelae in women with abnormal cervical cytology. However, these may have limited applicability because they include questions relating to distress specifically due to colposcopy or gynaecological examination, which may not be relevant to women managed in other ways, for example, by cytological surveillance.

The existence of an appropriate instrument to measure psychosocial burden in women with low-grade abnormalities (irrespective of the way in which they are managed) would allow a better estimation of the magnitude of the problem; facilitate comparison of the psychosocial burden associated with different managements strategies; and enable the evaluation of any interventions designed to reduce the psychosocial burden of receiving an abnormal cervical cytology result or management thereof.

Nested within the UK Cervical Screening Programmes, the TOMBOLA (Trial Of Management of Borderline and Other Low-grade Abnormalities) trial was a randomised controlled trial (RCT) which aimed to clarify the most effective and cost-effective management strategy for women with low-grade abnormal cervical cytology [17]. One component of the trial was the comparison of the psychosocial burden between women in the different management arms, immediate colposcopy versus cytological surveillance.

A number of previously validated questionnaires, including the Hospital Anxiety and Depression Scale (HADS) [18] and the EuroQoL EQ-5D-3 L [19], were used in TOMBOLA to measure women’s responses to having received a low-grade abnormal cervical cytology result and their subsequent management. However, no existing questionnaire could be identified to assess adequately the full spectrum of potential psychosocial consequences or one which was applicable to women managed in different ways.

The Process and Outcome Specific Measure (POSM) was therefore developed within TOMBOLA, to address this gap [20]. The POSM is a short, easy-to-use, 14 item questionnaire which can assess the psychosocial impact of receiving an abnormal cytology result and the impact of different management strategies. The POSM was specifically designed to capture aspects of women’s responses to an abnormal cytology result that would be sensitive to different processes of management. Although not originally designed to be reported as a single score, or a series of sub-scores, early pilot work [20] based on a small number of participants suggested that the POSM may have a suitable level of reliability to be reported as a scale.

This paper reports the results of an exploratory factor analysis and reliability analysis of the POSM questions utilising the full trial dataset, which included 3331 women. We aimed to explore possible latent factors in the pool of POSM questions and reliability of these factors in order to better inform future use and reporting of the POSM questions.

## Methods

### The TOMBOLA trial and participants

The TOMBOLA trial design has been described in detail elsewhere [17]. Briefly, eligible women were aged 20–59 years, had received a low-grade abnormal cervical cytology result (BNC or mild dyskaryosis) between October 1999 and October 2002 from a routine cervical screening test taken as part of the NHS Cervical Screening Programmes, lived in the Grampian or Tayside areas of Scotland or the Nottingham area of England, and had not had more than one additional BNC result in the previous three years. Women were not eligible if they had previous treatment for proven or suspected cervical lesions, or were pregnant at the time of recruitment. Eligible women were sent an information leaflet and a letter inviting them to a recruitment clinic, and were recruited on average 8 weeks after having the abnormal test. All women recruited from February 2001 onwards were included in the psychosocial component of the trial, and therefore eligible for this analysis.

### Measures

At the recruitment clinic, and after giving informed consent, women completed a socio-demographic and lifestyle questionnaire, and baseline psychosocial questionnaire. The psychosocial questionnaire included the POSM, the HADS and the EuroQoL EQ-5D-3 L.

The 14-items of the POSM related to: feeling well enough informed about the abnormal result; information received having addressed concerns; worries about health, cancer, next test showing changes, having sex and future fertility; delaying pregnancy; changes to feelings about self and to sex life; satisfaction with support, beliefs about cervical screening, future screening intentions, and perceived risk of developing cervical cancer. Each question had between five and seven response options on a Likert scale. Responses ranged from “Strongly agree” to “Strongly disagree” for most questions; from “Strongly for the better” to “Strongly for the worse” for the two questions relating to change; and from “Very much lower than average” to “Very much higher than average” for the perceived risk question. In addition, there were two filter questions relating to intention to have children and sexual activity, which allow women to skip questions which are not relevant to them. A copy of the POSM questions is included as an additional file (Additional file 1).

We developed two slightly different versions of the POSM. In the version administered at the recruitment clinic, the questions were framed to refer to the period between receiving the abnormal result and completing the questionnaire. The alternative version was developed for administration during follow-up and women received it by post at 12, 18, 24 and 30 months after recruitment. In this version the time frame for each question referred to the previous month and the question relating to the extent to which information received having addressed concerns women had about their smear result since the abnormal cytology result was omitted.

The HADS, was originally developed to screen for clinically significant levels of anxiety and depression in a medical outpatient setting, but has since been found to have acceptable reliability for use in primary care [21]. The HADS is a self reported questionnaire which consists of 14 items on two sub-scales, with seven measuring anxiety and seven measuring depression. Items are scored on a four point scale from 0 to 3, to yield a score out of 21 for each sub-scale. Scores are generally used to categorise subjects on each sub-scale into non-cases (0–7), possible cases (8–10), and probable cases (≥11) [18,21]. Questions refer to the previous week.

The EuroQoL EQ-5D-3 L [19] is a widely used generic measure of health related quality of life. This analysis uses the EQ-5D visual analogue scale (VAS), which consists of a 20 cm scale numbered 0–100. Respondents are asked to mark on the scale how they rate their health that day.

### Selection of questions for factor analysis

An initial review of the questions included in the POSM was undertaken in order to select a pool of questions which would be applicable to all participants and all versions of the POSM. To this end, some questions were deleted from the item pool before factor analysis.

One question (‘The information I have received has answered the concerns I have had about my smear result’) was not considered for inclusion in the factor analysis since it was only included in the version of the POSM used at baseline, and not included in the follow-up questionnaire.

Items were assessed on their face validity, i.e. to the extent to which they were related to the psychosocial burden of receiving an abnormal cervical cytology result. After discussion within the research team, it was decided that all but three items were related to psychosocial burden. The three items which were not related to psychosocial burden were deleted from the item pool and not considered for this analysis. These items were: “What do you feel your chances of getting cervical cancer are compared to other women”, “I believe that having regular smears reduces my risk of getting cervical cancer “and “I intend to continue having regular smears”.

Three of the remaining questions were only answered by particular groups of women. Two of these questions were only answered by women who were sexually active at the time of completing the questionnaire and one of these only by women intending to have children in the future. These three questions were also deleted from the item pool and not considered for factor analysis. This left 7 questions, henceforth referred to as “core” questions.

### Factor and reliability analysis

The initial analysis included data from 3331 women who completed the baseline POSM at recruitment. We conducted exploratory factor analysis on the core questions in order to identify any scales within the POSM questionnaire. We made no prior assumptions about any possible factor structure.

An inter-item correlation matrix of responses to the core POSM questions was prepared. Items were assessed for inclusion in the factor analysis on the basis of sufficient correlation (r > 0.2) with at least one other item.

The Kaiser-Meyer-Olkin (KMO) test and Bartlett’s test of sphericity were conducted to test whether there was sufficient common variance and correlation between core questions to carry out principal components analysis. According to convention [22], a minimum level of 0.5 was used for the KMO test to indicate sufficient common variance.

Both Cattell’s Scree plot method [22] and Kaiser’s criterion (retention of factors with eigenvalues greater than one) [23] were used to determine the number of factors to extract. Factors were then extracted using principal components analysis and rotated with varimax rotation [24].

The internal consistency reliability of the resultant factors was assessed using Cronbach’s alpha (C_α_). Item-total correlations were calculated in order to assess whether the items within each factor behaved consistently. Following standard practice [25], a correlation of over 0.2 was considered acceptable. Spearman’s correlation coefficient was calculated between items and each of the factors. We calculated corrected item-total correlations by calculating correlations between items and total scores for each factor excluding that item. We assessed discriminant validity by correlating individual items with the EQ-5D visual analogue scale (VAS) and the anxiety and depression sub-scales of the HADS. We assessed whether the POSM measured something distinct from health-related quality of life and anxiety and depression by correlating the factor scores with the HADS and EQ-5D VAS scores: in this event, it would be expected that items from the POSM would correlate more strongly with factor scores that they belong to than with the EQ-5D VAS score or the HADS sub-scale scores.

### Scoring

A scoring scheme was devised for the core questions such that a high score indicated greater psychosocial burden. Since the number of response options differed between the POSM items, scores were standardised. For each question, response categories were given a raw score, ranging from 1 to 6 (1 to 7 for the single question which had a central neutral response option). The raw score for each question was multiplied by 100 and divided by the maximum possible raw score for that question. Item responses for each question were thus standardised to be scored out of 100.

In order to obtain factor scores, standardised scores for the questions included in that factor were summed and divided by the number of items within the factor. Thus, scores for each of the factors were out of 100. To have a score for a factor, women had to answer all questions which form that factor.

### Sensitivity analysis

In order to assess the effect of excluding items which were judged to have low face validity, we performed a further factor analysis of the baseline data, this time including these items. Reliability analysis was then repeated.

A series of further factor analyses were performed using data from the questionnaires completed by women at 12 (n = 2181), 18 (n = 1993), 24 (n = 1880) and 30 (n = 1737) months post-recruitment. These analyses were then repeated at each time point for each trial arm separately. The results of these analyses were compared to that from the analysis of the baseline dataset to assess the temporal stability of the factor structure.

Since the POSM has been used as single scale in the past [20], we assessed the reliability of including the items in one score. We used the core questions, as these questions were relevant to all women and all versions of the POSM, and also all related to psychosocial burden. We constructed an “overall score” out of 100 in a similar way to the factor scores. We then calculated corrected item total correlations and Cronbach’s alpha in the same way as before.

### Details of ethical approval

Ethical approval was obtained from the Joint Research Ethics Committee of NHS Grampian and the University of Aberdeen (Reference 970072), the Tayside Committee on Medical Research Ethics (170/99) and the Nottingham Research Ethics Committee (PA129701).

## Results

### Characteristics of participants

The sociodemographic characteristics of the 3331 women and included in the factor analysis of baseline data are shown in Table 1.

**Table 1:** **Sociodemographic characteristics of women included in this study**

**Characteristic**	**N**	**%**
**Age group (n = 3331)**		
20-29	1440	43.2
30-39	887	26.6
40-49	708	21.3
50-59	296	8.9
**Ethnic group (N = 3318; missing = 13)**		
White	3180	95.8
Non-white	138	4.2
**Trial centre (N = 3331)**		
1	1068	32.1
2	834	25.0
3	1429	42.9
**Employment status (N = 3323; missing = 8)**		
Full-time paid employment	1663	50.0
Part-time paid employment	771	23.2
Student	307	9.2
Not in paid employment	582	17.5
**Carstairs quintile (N = 3331)**		
1 (Least deprived)	469	14.1
2	623	18.7
3	533	16.0
4	877	26.3
5 (Most deprived)	829	24.9
**Marital status (N = 3298; missing = 33)**		
Married or cohabiting	1829	55.5
Divorced, separated or widowed	445	13.5
Single	1024	31.0
**Reproductive history (N = 3297; missing = 34)**		
Never pregnant	1119	33.9
Ever pregnant	2178	66.1
**Cervical screening history (N = 3331)**		
No previous borderline nuclear changes	3022	90.7
Previous borderline nuclear changes	309	9.3
**Recruitment cytology test result (N = 3331)**		
Mild dyskaryosis	892	26.8
Borderline nuclear changes	2439	73.2
**Post school education and training (N = 3319; missing = 12)**		
None	885	26.7
Qualification through work	660	19.9
Qualification other than degree from college/university	953	28.7
Degree from college/university	821	24.7
**Currently using oral contraceptive (N = 3325; missing = 6)**		
No	2182	65.6
Yes	1143	34.4
**Physical activity outside work (N = 3291; missing = 40)**		
<Once per week	1298	39.4
1-3 times per week	781	23.7
>3 times per week	1212	36.8
**Smoking status (N = 3302; missing = 29)**		
Never Smoker	1575	47.7
Ex-smoker	567	17.2
Current Smoker	1160	35.1

### Responses

The responses to the POSM questions are summarised in Additional file 1. In general, the level of missing responses was low. The exception to this was 14.8% for the question “since receiving my smear result my sex life has changed”. Five of the items showed significant clustering of responses on single response options, including four which had been removed from the main pool of questions for this analysis: “I intend to continue having regular smears”; “I believe that having regular smears reduces my risk of getting cervical cancer”; “What do you feel your chances of getting cervical cancer are compared to other women”; and “Since getting my smear result my sex life has changed”. One was from the pool of remaining questions: “Since getting my smear result the way I feel about myself has changed”.

### Factor structure – baseline data

The Kaiser-Meyer-Olkin (KMO) measure for the core questions was 0.74, and Bartlett’s test of sphericity (p < 0.001) indicated that common variance between items was sufficient to carry out a principal components analysis.

### Factor analysis

Kaiser’s criterion and Cattel’s scree plot both suggested the extraction of two factors. The rotated factor structure of these factors is displayed in Table 2; values under 0.4 have been suppressed for clarity. The two extracted factors explained 54.7% of the variance from the original data.

**Table 2:** **Factor structure of the core POSM questions**

**POSM question**	**Worry factor**	**Information-Support factor**
Since getting my smear result I have been worried that I may have cervical cancer.	0.846	
Since getting my smear result I have been worried about my general health.	0.819	
Since getting my smear result I have been worried that my next smear will show changes to the cells.	0.814	
Since getting my smear result I have been worried about having sex.	0.581	
In general I feel well enough informed about what my smear result means.		0.778
Since getting my smear result I have been satisfied with the support I have had from other people.		0.767
Since getting my smear result the way I feel about myself has changed.		0.445

Factor loadings under 0.4 have been suppressed for clarity.

All of the items loaded onto a factor, and none of the items loaded onto more than one factor. The items that were associated with each other suggested that the first factor represented worries (hereafter referred to as the Worry factor) and the second factor represented information and support (henceforth Information-Support factor). In addition to questions about feeling well enough informed and being satisfied with support from other people, this second factor also included the question on changes in how women felt about themselves. The low loading of this latter question (0.445) indicates that, although it fits better in this factor than elsewhere, its association with this factor is less strong than the other two questions.

### Reliability

Corrected item-total correlations are shown in Table 3. The item-total correlations between constituent items and the Worry score were moderate to high (between 0.405 and 0.659). The item-total correlation for two of the Information-Support score questions (“In general I feel well enough informed about what my smear result means” and “Since getting my smear result I have generally been satisfied with the support I have had from other people”) was moderate. However the item total correlation for the other question (“Since getting my smear result the way I feel about myself has changed”) was low.

**Table 3:** **POSM item-total correlations and correlations with the HADS scales and the EQ-5D VAS**

**POSM question (Abbreviated)**	**Worry factor**	**Information-Support factor**	**Overall score**	**HADS anxiety**	**HADS depression**	**EQ-5D VAS**
**Worried that I may have cervical cancer**	**0.659**	0.101	**0.587**	0.410	0.254	−0.131
**Worried about general health**	**0.636**	0.061	**0.560**	0.412	0.278	−0.198
**Worried next smear will show changes to the cells**	**0.618**	0.081	**0.550**	0.382	0.200	−0.132
**Worried about having sex**	**0.405**	0.083	**0.385**	0.311	0.236	−0.142
**Well enough informed about what smear result means**	0.122	0.326*	**0.205**	0.109	0.087	−0.072
**Satisfied with support from other people**	0.049	0.298*	**0.147**	0.085	0.124	−0.086
**Way I feel about myself has changed**	0.238	**0.161**	**0.187**	0.196	0.149	−0.075

Items which form a part of these scores are indicated in bold and are corrected item-total correlations.

*0.319 when item “way I feel about myself has changed” is removed from Information-Support factor score.

The Worry factor had good reliability (C_α_ = 0.769). The reliability of the Information-Support factor was poorer (C_α_ = 0.430). Removal of the question relating to changes in the way women felt about themselves, increased reliability slightly (C_α_ = 0.482).

Table 4 shows the correlations between the Worry and Information-Support factors, the overall score and the HADS and EQ-5D VAS scores. These results indicate that there is some overlap between the Worry factor and HADS anxiety. However, the correlations between the factor scores and the HADS depression score, and EQ-5D VAS scores were lower.

**Table 4:** **Correlations between the POSM scores and the HADS and EQ-5D VAS scores**

	**HADS anxiety**	**HADS depression**	**EQ-5D VAS**
Worry	0.487	0.316	−0.192
Information-Support	0.145	0.140	−0.111
Overall score	0.486	0.330	−0.200

### Sensitivity analysis

Cronbach’s alpha for the overall score for the core questions was 0.668, slightly less than the acceptable level. Most of the correlations between individual questions and the overall score were acceptable (0.205-0.587). However two questions had correlations of less than 0.2; these related to changes in the way women felt about themselves, and satisfaction with support from other people. One question, changes in the way women felt about themselves, correlated more highly with the HADS anxiety subscale than with the overall score. Correlations between the overall score and the HADS and EQ-5D VAS scores followed a similar pattern to the Worry factor (Table 4).

When we included the questions we had previously removed because of low face validity, the previous two factors (Worry and Information-Support) did not change. One of the new questions “What do you feel your chances of getting cervical cancer are compared to other women” did not load onto any factor. The other two questions “I intend to continue having regular smears” and “I believe that having regular smears reduces my risk of getting cancer” both loaded onto a new, third factor. This factor had very low reliability (C_α_ = 0.217).

Analysis of data collected at 12, 18, 24 and 30 months post-recruitment indicated that both the Worry and Information-support factors were stable over time, and that there was no difference in factor structure between trial arms, i.e. the same questions loaded onto the same factors (data not shown).

## Discussion

### Summary of results

Psychosocial sequelae are an important consequence of both the receipt of an abnormal cervical cytology result and the subsequent management of the abnormality [13]. The POSM was developed to measure these psychosocial sequelae.

We investigated a pool of POSM questions to identify possible latent factors, and assessed the ability of these factors to act as scales. Factor analysis revealed that that there were two latent factors. These related to: (1) Worry (four questions) and (2) Satisfaction with information and support (three questions).

We removed some questions from the pool of POSM questions to be entered into the factor analysis. Three of the questions were removed as it was felt that they did not relate to psychosocial distress. This decision was borne out in the sensitivity analysis which showed that these items, when added to the factor analysis, did not change the structure of the initial two factors, and also did not form a factor with appreciable reliability. In addition, all three of these items were among the five items which showed significant clustering of responses. We also removed three questions from the pool because they were only answered by some women. This decision was driven by the desire to explore the possibility of finding a useful summary score, or scores, within the POSM items which would be relevant to all women the POSM was administered to.

The reliability of the Worry factor was supported by reliability assessment. However, the reliability measures for the Information-Support factor were somewhat lower, but considering the small number of items in this factor (n = 3) this was not surprising. In the additional analysis, the reliability of the overall score based on the core questions fell just below the pre-defined acceptable level of reliability.

The discriminant validity of the POSM scores was demonstrated by the correlations between factor scores and the overall score and the EQ-5D VAS and HADS scores. These results indicated that, in general, the POSM core questions measured constructs which were distinct from anxiety and depression (as measured by the HADS) and self-perceived health-related quality of life (as measured by the EQ-5D VAS). Thus, the POSM achieved its aim in measuring the psychosocial consequences of receiving an abnormal cytology result, and the management thereof, beyond that measured by existing questionnaires.

These results also indicate that higher scores (more distress) on the scales assessed here were associated with lower health-related quality of life (lower EQ-5D VAS scores). It also appears that the Worry factor score is related to HADS measured anxiety, and to a lesser extent, HADS measured depression. The correlations between the overall score and the other measures followed a similar pattern to the Worry factor score. This was not surprising as these two scores differed only by three items.

The temporal stability of the POSM factor structure was confirmed by further factor analysis of responses to the POSM using 12, 18, 24 and 30 month follow up datasets from the TOMBOLA trial.

### Scoring the POSM

The method used here for scoring the POSM differs slightly from the method used during the original pilot study [20]. The method presented here is based on the results of the factor analysis, analysis of a larger dataset and further thinking about how the POSM could be used in other populations. Standardising the scores for individual questions, and in the summing of these, allows one to compare total scores for women who may have completed different numbers of the core questions. This is useful since, as with all questionnaires, a small number of respondents may not chose to answer all individual questions.

### Strengths and weaknesses

One potential weakness of this study is the age of the data, which were collected between 1999 and 2003 for the baseline analysis. However, we have no reason to believe that psychosocial distress relating to cervical screening has changed in such a way as to impact on the psychometric properties of this instrument. Also, the data used in this study were obtained from women who had consented to take part in an RCT and, as a result, may have differed from women in the general screening population. However, the sample used for both the original POSM development and the analysis presented in this paper was drawn from the UK cervical screening population and the trial design was population-based (i.e. all eligible women in the relevant areas were invited to participate). This is a strength compared to other questionnaires [15], which have used sampling strategies which over-represented some groups used to generate and validate questionnaire items. This study also benefits from a large sample size, which was in excess of 3300 women. A further advantage of the POSM is that it is not management-specific, that is, it does not contain items which are only relevant to women who had received a particular intervention as a result of their abnormal cervical cytology; this is in contrast to other similar measures [14–16]. Furthermore, the POSM has been designed such that it measures the psychosocial burden of both initial receipt of an abnormal cytology result and its subsequent follow up. In practice, this means that the POSM may be used to compare the psychosocial effects of receiving an abnormal cervical cytology result between women who subsequently underwent different clinical follow-up. It is interesting, but perhaps unsurprising, that the Worry factor extracted from the POSM incorporates some similar questions to those included in the “worries and concerns” scale of the HPV Impact Profile, for example about abnormal smear test results and cervical cancer [16].

### Future use of the POSM

Although the POSM was developed for use in women who have received a low-grade abnormal cytology result in the context of the UK screening population, it has the potential to be used in other populations, such as in women with higher grade cytology or with precancerous or cancerous lesions, and those with other gynaecological disorders. Indeed, the POSM has recently been used in women with microinvasive cancer of the cervix [26], in women who have been referred for colposcopy after either a low or high-grade abnormal cytology result [27] and in women with vulval intraepithelial neoplasia [28].

Cervical screening and management are changing, with an increasing focus on testing for infection with high-risk types of human papillomavirus (HPV). The nature of the questions in the POSM suggests that there is no impediment to its use in women who have had HPV tests in addition to, or instead of, cytology tests. For example, following the introduction of HPV triage in the NHS in England [29], there may be scope for using the POSM in measuring the psychosocial effects of a positive HPV test. Alternatively, following the adoption of cytology and HPV co-testing for primary screening in the USA [30,31] and in the Netherlands [32], it may be possible to use the POSM to compare the psychosocial impact of a positive HPV test (and negative cytology test), an abnormal cytology test (and negative HPV test), or positive cytology and HPV tests.

For future use of the POSM, we recommend that the pool of seven core questions tested here be administered to all women. These questions are relevant to all women. The use of the three supplementary questions (those relating to women who are sexually active and/or planning on having children) will depend on their relevance to the research question and the sociodemographic characteristics of the study population. The same applies to the remaining questions (those which were qualitatively different from the core and supplementary questions because they concerned perceptions of risk and intended behaviour). In reporting the POSM, researchers may choose to report women’s responses to individual questions (if, for example, specific issues such as worries about cervical cancer are of particular interest). As this analysis suggests, responses to the four relevant questions which are part of the worry factor may be reported as a worry score in addition to reporting responses to the remaining individual items which do not form the worry factor. Alternatively, researchers may, for pragmatic reasons, wish to report an overall score for the seven questions relevant to all women. The psychometric properties of this overall score are reported here for guidance.

## Conclusions

This study has demonstrated two latent factors in a sub-set of POSM questions relating to psychosocial burden. Reliability analysis has shown that one of these factors has suitable psychometric properties to be used as a scale to measure worries related to cervical screening. This analysis will inform its future use in this population and in other related contexts.

## Additional file

Additional file 1:
**Responses to POSM questions.**

## Abbreviations

BNC: Borderline nuclear changes
EQ-5D: Euroqol -5D
HADS: Hospital anxiety and depression scale
HPV: Human papillomavirus
KMO: Kaiser-meyer-olkin
RCT: Randomised controlled trial
TOMBOLA: Trial of management of borderline and other low-grade abnormal smears
VAS: Visual analogue scale
