# Supplementary material for: Measuring the psychosocial burden in women with low-grade abnormal cervical cytology in the TOMBOLA trial: psychometric properties of the Process and Outcome Specific Measure (POSM)
Source: Health Qual Life Outcomes. 2014 Nov 12;12:154. doi: 10.1186/s12955-014-0154-8 (PMC4252018; doi:10.1186/s12955-014-0154-8)
Supplement: Additional file 1: — Responses to POSM questions. [file 12955_2014_154_MOESM1_ESM.docx]

**Additional file 1.** Responses to POSM questions

| **Question** | **Frequency n (%)** |
| --- | --- |
| **In general I feel well enough informed about what my smear result means (N=3399)** |  |
| Strongly agree | 1552 (45.6) |
| Moderately agree | 1118 (32.9) |
| Slightly agree | 282 (8.3) |
| Slightly disagree | 157 (4.6) |
| Moderately disagree | 113 (3.3) |
| Strongly disagree | 83 (2.4) |
| Missing | 94 (2.8) |
|  |  |
| **Since getting my smear result I have been worried about my general health (N=3399)** |  |
| Strongly agree | 419 (12.3) |
| Moderately agree | 376 (11.1) |
| Slightly agree | 188 (5.5) |
| Slightly disagree | 1128 (33.2) |
| Moderately disagree | 751 (22.1) |
| Strongly disagree | 438 (23.9) |
| Missing | 99 (2.9) |
|  |  |
| **Since getting my smear result the way I feel about myself has changed (N=3399)** |  |
| Strongly for the better | 75 (2.2) |
| Moderately for the better | 104 (3.1) |
| Slightly for the better | 129 (3.8) |
| Neither better nor worse | 2133 (62.8) |
| Slightly for the worse | 710 (20.9) |
| Moderately for the worse | 94 (2.8) |
| Strongly for the worse | 39 (1.2) |
| Missing | 115 (3.4) |
|  |  |
| **Since getting my smear result I have been worried that my next smear will show changes to the cells (N=3399)** |  |
| Strongly agree | 119 (3.5) |
| Moderately agree | 116 (3.4) |
| Slightly agree | 121 (3.6) |
| Slightly disagree | 1239 (36.5) |
| Moderately disagree | 874 (25.7) |
| Strongly disagree | 827 (24.3) |
| Missing | 103 (3.0) |
|  |  |
| **Since getting my smear result I have been worried that I may have cervical cancer (N=3399)** |  |
| Strongly agree | 425 (12.5) |
| Moderately agree | 305 (9.0) |
| Slightly agree | 317 (9.33) |
| Slightly disagree | 1188 (35.0) |
| Moderately disagree | 532 (15.7) |
| Strongly disagree | 525 (15.5) |
| Missing | 107 (3.1) |
|  |  |
| **Since getting my smear result I have been worried about my ability to have children in the future (N=1239)** |  |
| Strongly agree | 178 (14.4) |
| Moderately agree | 170 (13.7) |
| Slightly agree | 409 (33.0) |
| Slightly disagree | 138 (11.1) |
| Moderately disagree | 120 (9.7) |
| Strongly disagree | 215 (17.4) |
| Missing | 9 (0.7) |
|  |  |
| **Since getting my smear result I have decided to delay getting pregnant (N=1239)** |  |
| Not applicable | 536 (43.3) |
| Strongly agree | 65 (5.3) |
| Moderately agree | 38 (3.1) |
| Slightly agree | 95 (7.7) |
| Slightly disagree | 89 (7.2) |
| Moderately disagree | 89 (7.2) |
| Strongly disagree | 305 (24.6) |
| Missing | 22 (1.8) |
|  |  |
| **Since getting my smear result I have been worried about having sex (N=3399)** |  |
| Strongly agree | 109 (3.2) |
| Moderately agree | 226 (6.7) |
| Slightly agree | 564 (16.6) |
| Slightly disagree | 332 (9.8) |
| Moderately disagree | 439 (12.9) |
| Strongly disagree | 1559 (45.9) |
| Missing | 170 (5.0) |
|  |  |
| **Since getting my smear result my sex life has changed** |  |
| Strongly for the better | 17 (0.5) |
| Moderately for the better | 27 (0.8) |
| Slightly for the better | 27 (0.8) |
| Neither better nor worse | 2550 (75.0) |
| Slightly for the worse | 209 (6.2) |
| Moderately for the worse | 40 (1.2) |
| Strongly for the worse | 25 (0.7) |
| Missing | 504 (14.8) |
|  |  |
| **I intend to continue having regular smears (N=3399)** |  |
| Strongly agree | 3113 (91.6) |
| Moderately agree | 142 (4.2) |
| Slightly agree | 28 (0.8) |
| Slightly disagree | 2 (0.1) |
| Moderately disagree | 0 (0) |
| Strongly disagree | 8 (0.2) |
| Missing | 106 (3.1) |
|  |  |
| **I believe that having regular smears reduces my risk of getting cancer (N=3399)** |  |
| Strongly agree | 2653 (78.1) |
| Moderately agree | 377 (11.1) |
| Slightly agree | 106 (3.1) |
| Slightly disagree | 30 (0.9) |
| Moderately disagree | 22 (0.7) |
| Strongly disagree | 55 (1.6) |
| Don’t know | 51 (1.5) |
| Missing | 105 (3.1) |
|  |  |
| **What do you feel your chances of getting cervical cancer are compared to other women (N=3399)** |  |
| Very much lower than average | 90 (2.7) |
| Somewhat lower than average | 225 (6.6) |
| Average | 2345 (69.0) |
| Somewhat higher than average | 548 (16.1) |
| Very much higher than average | 42 (1.2) |
| Missing | 149 (4.4) |
|  |  |
| **Since getting my smear result I have generally been satisfied with the support I have had from other people** |  |
| Strongly agree | 1134 (33.4) |
| Moderately agree | 1130 (33.3) |
| Slightly agree | 685 (20.2) |
| Slightly disagree | 152 (4.5) |
| Moderately disagree | 67 (2.0) |
| Strongly disagree | 49 (1.4) |
| Missing | 182 (5.4) |
